# Supplementary material for: Inter-Row Grassing Reshapes Nitrogen Cycling in Peach Orchards by Influencing Microbial Pathways in the Rhizosphere
Source: Microorganisms. 2025 Dec 5;13(12):2770. doi: 10.3390/microorganisms13122770 (PMC12735878; doi:10.3390/microorganisms13122770)
Supplement: Supplementary file 1 [file microorganisms-13-02770-s001.zip › microorganisms-3989831-supplementary.pdf]

Class	Samples	Clean reads	Percent in raw reads (%)[a]	Contigs	ORFs[b]	N50(bp) [c]	N90(bp) [d]
Grass rhizosphere	CK1	79023198	98.02	558271	638223	497	334
	CK2	79173232	98.01	618336	701980	492	333
	CK3	85681186	98.14	622538	697577	482	332
	PR1	90318314	98.66	1005530	1163024	492	335
	PR2	84658912	98.56	712764	804100	471	331
	PR3	84444720	98.55	709819	804449	466	330
	TR1	83807408	98.63	1025222	1195996	503	336
	TR2	84224530	98.56	840612	958724	489	333
	TR3	90328452	98.41	931812	1080631	504	336
	TPR1_P	85510910	98.28	903384	1069919	517	339
	TPR2_P	86573658	98.03	990825	1182114	524	339
	TPR3_P	82885590	98.31	937014	1104366	505	337
	TPR1_T	79830336	98.20	563513	647473	488	333
	TPR2_T	91451712	98.63	957726	1084053	481	332
	TPR3_T	86992296	98.46	863312	980991	481	332
Tree rhizosphere	CK1	85680692	98.19	965906	1177763	547	341
	CK2	78245296	98.16	552932	648463	514	336
	CK3	87116466	98.34	851477	1012600	508	336
	PR1	85599428	98.65	771609	905924	513	336
	PR2	91985314	98.69	895143	1054789	512	336
	PR3	84192562	98.66	762248	881154	493	334
	TR1	83210610	98.68	801823	954194	520	337
	TR2	93395574	98.62	863229	1028640	521	337
	TR3	84798828	98.56	681187	762347	481	332
	TPR1	85450048	98.54	1096101	1333553	538	341
	TPR2	89569124	98.14	1031113	1369871	725	363
	TPR3	89636438	98.66	743397	852125	485	332

Table S1 Basic information of metagenomic sequencing.

[a]The percentage of clean read in its corresponding raw read.

[b]ORFs, Open reading frames.

[c]N50 represents the length of the contig overlapping the midpoint of the length-order concatenation of contigs.

[d]N90 represents the length of the contig overlapping the ninety percent of the length-order concatenation of contigs.

CK, clean tillage; PR, perennial ryegrass; TR, *Trifolium repens*; TPR, mixed sowing of perennial ryegrass and *Trifolium repens*; TPR_P, perennial ryegrass in mixed sowing; TPR_T, *Trifolium repens* in mixed sowing.

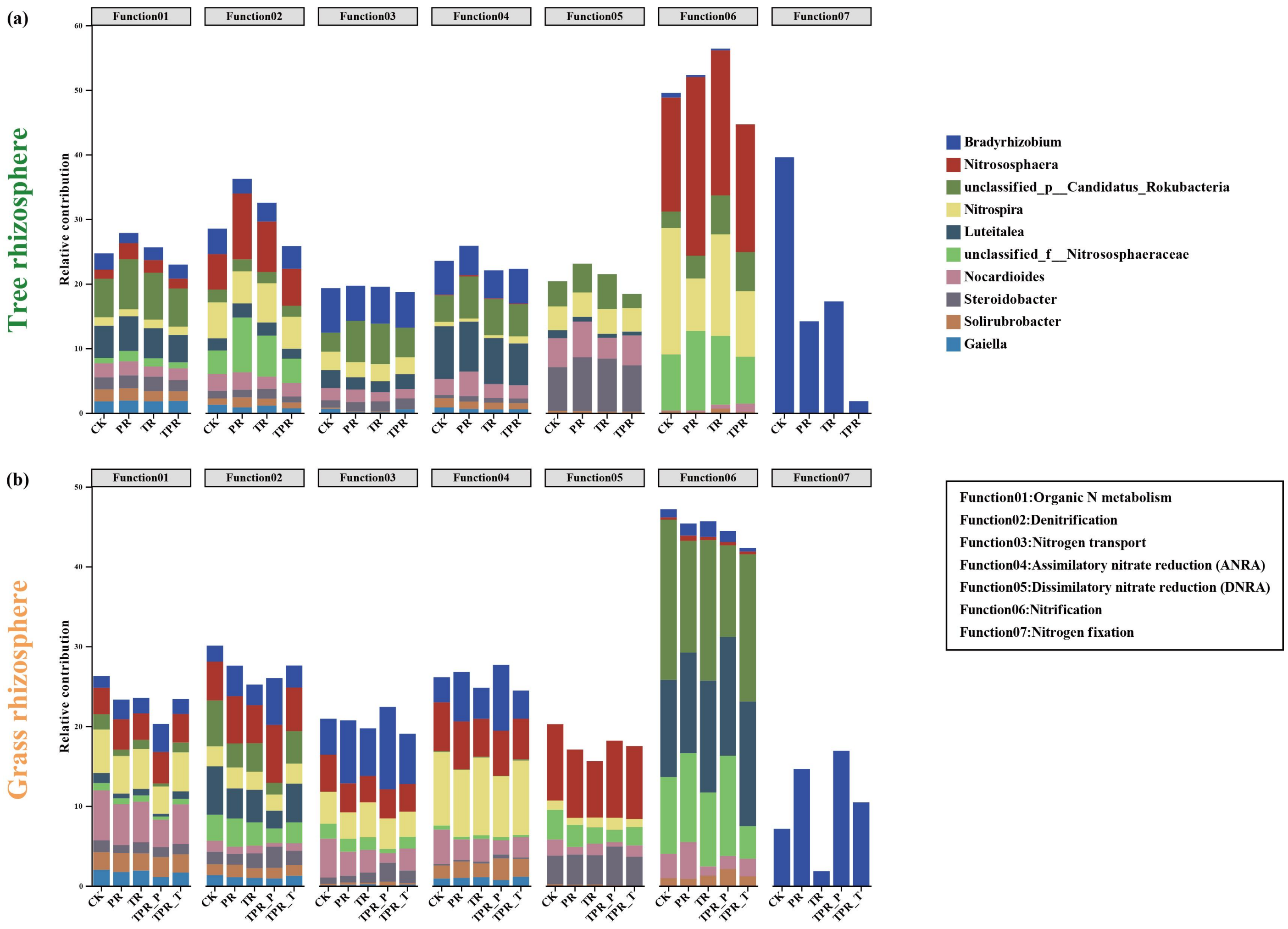

Figure S1. Relative contributions of dominant nitrogen-cycling microorganisms to different nitrogen transformation processes in (a) tree rhizosphere and (b) grass rhizosphere under various inter-row grassing treatments. CK, clean tillage; PR, perennial ryegrass; TR, *Trifolium repens*; TPR, mixed sowing of perennial ryegrass and *Trifolium repens*; TPR_P, perennial ryegrass in mixed sowing; TPR_T, *Trifolium repens* in mixed sowing.

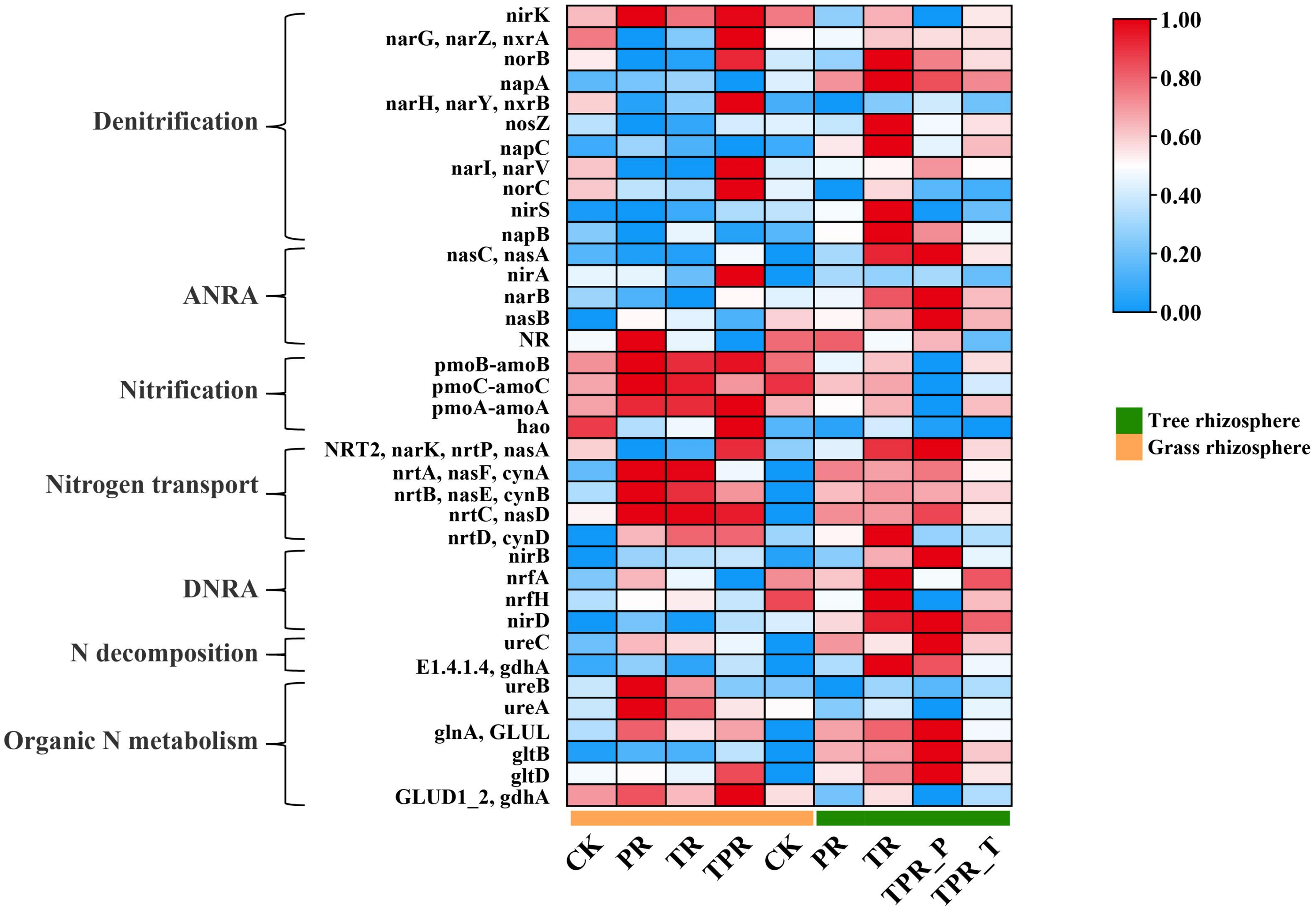

Figure S2. Heatmap showing the relative abundances of functional genes involved in major nitrogen-cycling pathways under different inter-row grassing treatments. CK, clean tillage; PR, perennial ryegrass; TR, *Trifolium repens*; TPR, mixed sowing of perennial ryegrass and *Trifolium repens*; TPR_P, perennial ryegrass in mixed sowing; TPR_T, *Trifolium repens* in mixed sowing.

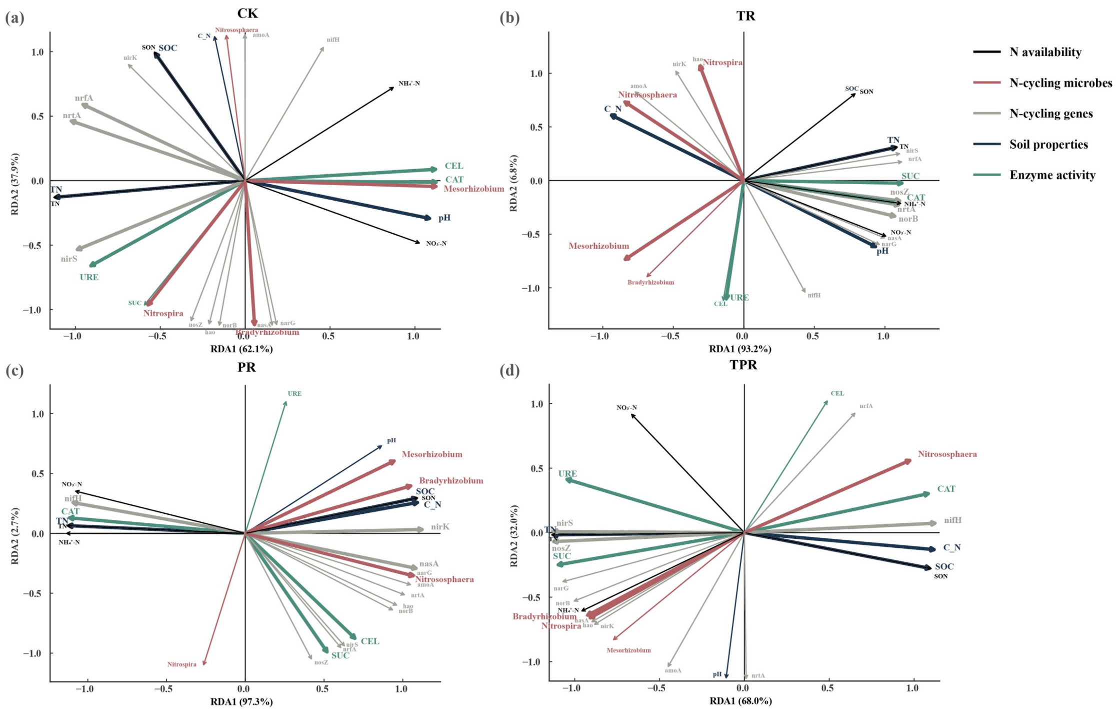

Figure S3. Redundancy analysis (RDA) showing the relationships between soil nitrogen availability and key environmental and biological factors under different inter-row grassing treatments: (a) CK (clean tillage), (b) TR (*Trifolium repens*), (c) PR (perennial ryegrass), and (d) TPR (mixed sowing of perennial ryegrass and *Trifolium repens*). Arrows represent nitrogen availability (black), nitrogen-cycling microorganisms (red), nitrogen-cycling genes (gray), soil physicochemical properties (navy), and enzyme activities (teal). Highlight arrows indicate stronger correlations between variables and nitrogen availability.
